# Supplementary material for: Stacking Model‐Based Classifiers for Dealing With Multiple Sets of Noisy Labels
Source: Biom J. 2025 Mar 12;67(2):e70042. doi: 10.1002/bimj.70042 (PMC11898607; doi:10.1002/bimj.70042)
Supplement: Supplementary file 1 — Supporting Information [file BIMJ-67-e70042-s001.zip › density-based-ensemble-model-main/competitors_real_data/netlab-master/netlab-master/help/gtmmag.htm]

Netlab Reference Manual gtmmag


# gtmmag

## Purpose

Magnification factors for a GTM

## Synopsis

```
mags = gtmmag(net, latentdata)
```

## Description

`mags = gtmmag(net, latentdata)` takes a GTM
structure `net`, and computes the magnification factors
for each point the latent space contained in `latentdata`.

## See Also

`gtm`, `gtmpost`, `gtmlmean`

---

**Pages:**
Index


---

Copyright (c) Ian T Nabney (1996-9)
